# Comparative analysis of oral microbiome in saliva samples of oral leukoplakia, proliferative leukoplakia and oral squamous cell carcinoma

**DOI:** 10.3389/froh.2025.1600090

**Published:** 2025-05-14

**Authors:** Rossella Intini, Sol Balsells, Leticia Bagan, Giulio Fortuna, Herve Sroussi, Jose Bagan

**Affiliations:** ^1^ Oral Medicine Unit, Stomatology Department, Valencia University, Valencia, Spain; ^2^ Statistical Advising Service, Fundació de Recerca Sant Joan de Déu, Esplugues de Llobregat, Spain.; ^3^ Department of Medicine, Surgery and Dentistry, University of Salerno, Salerno, Italy; ^4^ Division of Oral Medicine and Dentistry, Brigham and Women’s Hospital and Dana Farber Cancer Institute Department of Oral Medicine, Infection and Immunity, Harvard School of Dental Medicine, Boston, MA, United States; ^5^ Medicina Bucal Unit, Stomatology Department, Valencia University, Valencia, Spain; ^6^ Precancer and Oral Cancer Research Group, Valencia University, Valencia, Spain

**Keywords:** oral microbiome, oral leukoplakia, proliferative verrucous leukoplakia, oral squamous cell carcinoma, 16S rRNA sequencing, microbial diversity, dysbiosis, biomarkers

## Abstract

**Background:**

Oral potentially malignant disorders (OPMDs), including conventional leukoplakia (OL) and proliferative verrucous leukoplakia (PVL), have distinct risks of progression to oral squamous cell carcinoma (OSCC). A role of the oral microbiome in this transformation is increasingly recognized, but its contribution remains unclear.

**Objective:**

This study aimed to analyze and compare the oral microbiota in patients with OL, PVL, and OSCC using 16S rRNA gene sequencing of saliva samples to identify microbial signatures associated with disease progression and to uncover potential biomarkers that would justify an aggressive treatment of OPMDs.

**Methods:**

Sixty-six subjects with OPMDs were enrolled, comprising OL (*n* = 10), PVL (*n* = 28), and OSCC (*n* = 28). Saliva samples were collected, and DNA was extracted. The V3–V4 regions of the 16S rRNA gene were sequenced using the Illumina MiSeq platform. Bioinformatic analyses, including diversity assessments and taxonomic classification with the SILVA v138 database, were performed using QIIME2. Alpha diversity was evaluated with Chao1, Shannon, and Simpson indices, while beta diversity was assessed using Bray-Curtis and Jaccard distances.

**Results:**

PVL exhibited the highest species richness, followed by OL, with OSCC showing the lowest diversity. While alpha diversity differences among the groups were not statistically significant (*p* > 0.05), beta diversity revealed distinct microbial community structures between OL and both PVL and OSCC (*p* < 0.05), but not between PVL and OSCC. At the phylum level, *Firmicutes* predominated across all groups, with significantly higher *Actinobacteriota* levels in OL (*p* = 0.002).

**Conclusion:**

Distinct microbial patterns differentiate OL from PVL and OSCC, with OL being different from PVL and OSCC, suggesting progressive microbial dysbiosis in malignant transformation. These findings support the potential of oral microbiome profiling as a non-invasive diagnostic and prognostic tool in oral oncology and highlight the need for longitudinal studies to establish causal relationships.

## Introduction

Oral potentially malignant disorders (OPMDs) are defined as pathological abnormalities of the oral mucosa that carry a risk of progression to oral squamous cell carcinoma (OSCC) (1).

Among OPMDs, oral leukoplakia (OL) is the most prevalent, clinically defined as a white plaque of uncertain risk, having excluded other known diseases that carry no increased risk for cancer (2). OL can develop on any part of the oral mucosa, most frequently affecting the buccal mucosa and gingiva. However, lesions in high-risk locations, such as the floor of the mouth, ventrolateral tongue, and soft palate, are more likely to undergo cancer transformation (3). The malignant transformation rate of OL varies significantly across studies, with a systematic review estimating an overall transformation rate of 9.8% (4). Several factors influence this risk, including advanced age, female sex, lesion size, non-homogeneous appearance, anatomical location, and, most notably, the presence of epithelial dysplasia, which remains the strongest predictor of malignant progression (2, 5–7).

A more aggressive form of OPMD is proliferative verrucous leukoplakia (PVL), first described by Hansen et al. in 1985 (8). Since its initial characterization, PVL has been the subject of terminological debate, with alternative names such as *oral florid papillomatosis*, *proliferative multifocal leukoplakia*, and *multifocal verrucous leukoplakia* proposed in the literature (9–13). PVL predominantly affects older women, with diagnosis typically occurring in the sixth or seventh decade of life (13–15). Notably, unlike conventional leukoplakia, PVL is frequently observed in non-smokers and does not exhibit a significant association with alcohol consumption (13, 16). Over time, diagnostic criteria have evolved, with various classifications proposed by researchers, including Hansen, Cerero-Lapiedra et al., and Villa et al. (8, 13, 17) Pivotal to PVL diagnosis is the observation of the gradual, and relentless topographical and histopathological changes during its development (2). A hallmark of PVL is its relentless and progressive nature, both clinically and histopathologically. Initially, PVL may present as isolated leukoplakia lesions—flat, verrucous, or sometimes with a lichenoid appearance. However, it eventually spreads to multiple locations, either through the expansion of a single focus or the gradual merging of contiguous lesions (1, 4, 13).

The transition from OPMDs to OSCC is driven by cumulative genetic mutations, and while risk factors for both conditions overlap, not all OPMDs undergo malignant transformation (14). Nevertheless, they establish a microenvironment in which neoplasia is significantly more probable than in normal oral mucosa. Additionally, the presence of OPMDs is considered an indicator of future cancer risk, not only at the lesion site but also in clinically normal mucosa, where molecular alterations may already be present (1, 2). Early detection and excision of precancerous lesions are crucial in preventing cancer development, with studies showing that patients diagnosed with OPMDs tend to be, on average, five years younger than those diagnosed with OSCC (18). Importantly, failure to diagnose malignancies at an early stage can drastically reduce survival rates, with five-year survival for late-stage OSCC being less than 40% as opposed to 79.7% for patients with stage 1 OSCC (6, 19, 20).

In recent years, growing attention has been directed toward the role of the oral microbiome in OPMD progression and OSCC development. However, its exact involvement remains elusive. It is still unclear whether microbial alterations serve as a precursor event in oncogenesis—potentially actively contributing to malignant transformation—or if they are a late event derived from an opportunistic colonization in a tumor environment. This longstanding “chicken or the egg” question remains unresolved. The issue is complicated by multiple factors—such as smoking habits, diet, geographic location, and interindividual variability—continuously shaping the composition of the oral microbiota, making cross-study comparisons difficult (21). Despite these complexities, emerging evidence suggests that microbial dysbiosis may contribute to carcinogenesis in 7%–15% of oral cancer cases (22, 23). One proposed mechanism involves interactions between dysbiosis and the epithelial barrier, where an altered microbiome can promote immune system imbalance, disrupt mucosal integrity, and ultimately foster oncogenesis (24). Furthermore, dysbiosis has been implicated in later stages of carcinogenesis, potentially influencing tumor differentiation, immune evasion, and metastatic potential. However, pinpointing the exact microbial contributors to these processes remains challenging due in part to the oral cavity's highly specialized microenvironments (25).

Although the association between oral microbial imbalances and OSCC has been increasingly recognized, the precise contribution of different microbial taxa to OPMD initiation, progression, and malignant transformation remains poorly understood (26–28). Indeed, a fundamental question remains unanswered: *can microbial composition predict which OPMDs will transform into OSCC?* Identifying specific microbial shifts within lesions may enable the discovery of novel diagnostic and prognostic biomarkers, paving the way for microbiome-based therapeutic interventions targeting precancerous conditions (29, 30).

Given the limited knowledge pertaining to the role of dysbiosis in OPMD progression, this study aims to characterize the oral microbiome in patients with OL, PVL, and OSCC. Through 16S rRNA gene sequencing, we seek to determine whether specific microbial patterns differentiate these conditions, thus advancing our understanding of the potential microbiome's contributions to oral carcinogenesis. If microbial profiling can differentiate low-risk from high-risk OPMDs before histopathological alterations emerge, it could revolutionize early detection and targeted therapies, offering a powerful tool for precision medicine in oral oncology and potentially mitigating the risk of malignant transformation.

## Materials and methods

### Patients and samples

Sixty-six subjects were enrolled at the Stomatology and Maxillofacial Surgery Department of the General University Hospital of Valencia (2021–2023). Patients were assigned to three groups: 10 with OL [diagnosed through tissue biopsy and per the latest OPMD consensus by Warnakulasuriya et al. (1)]; 28 with PVL [diagnosed through tissue biopsy and per the criteria proposed by Villa et al. (13)]; and 28 with OSCC. None had undergone prior surgical treatment. Pregnant patients and under the age of 18 were excluded from the study.

Patients were instructed to fast for at least two hours before the procedure. None of the patients had taken any medication in the three months prior. Unstimulated whole saliva was collected in 15 ml tubes for five minutes via a funnel following the protocol of Navazesh et al. (31) To prevent blood contamination of the saliva, participants were instructed not to brush their teeth within 45 min prior to sample collection; saliva samples visibly contaminated with blood were discarded and recollected. The samples were then stored at −80°C until analysis.

### Library preparation and illumina sequencing

We aimed to characterize the bacterial populations in the afore-mentioned groups through the Next-generation sequencing of the V3–V4 region of 16S rRNA gene used as the taxonomic basis. According to Caporaso et al., 16S rRNA-based surveys are beneficial given since they can document unexplored biodiversity and characteristics of either whole communities or individual microbial taxa (32). DNA concentration was determined in the samples using a fluorimetric method using Quant- IT PicoGreen reagent in a FLUOROSCAN fluorimeter (Thermo Fisher). Variable V3 and V4 regions of the 16S rDNA gene were amplified following the 16S rDNA gene Metagenomic Sequencing Library Preparation Illumina protocol (Cod. 15044223 Rev. A). Gene-specific primers (PCR1_f: 5′ TCGTCGGCAGCGTCAGATGTGTATAAGAGACAGCCTACGGGNGGCWGCAG-3′; PCR1_r: 5′ GTCTCGTGGGCTCGGAGATGTGTATAAGAGACAGGACTACHVGGGTATCTAATCC-3′) containing Illumina adapter overhang nucleotide sequences were selected according to Klindworth et al. (33). After 16S rDNA gene amplification, the multiplexing step was performed using Nextera XT Index Kit. Multiplexing allows the sequencing of multiple samples simultaneously in the same run by adding unique “index” sequences (via the Nextera XT Index Kit) to each sample's DNA. This allows the Illumina sequencer to distinguish between different samples later on, even though they are sequenced together. We run 1 μl of the PCR product on a Bioanalyzer DNA 1,000 chip to verify the amplicons size (∼550 bp) on a Bioanalyzer (Agilent). After size verification the libraries were sequenced using a 2 × 301 pb paired-end run (MiSeq Reagent kit v3) on a MiSeq Sequencer according to manufacturer's instructions (Illumina).

### Bioinformatic data processing and analyses

Sequencing data were demultiplexed using Illumina bcl2fastq© program. Demultiplexed paired FASTQ sequences were imported into the QIIME2 v2023.2 platform for reads pre-procesing and microbiome analysis. Quality control was carried out using the DADA2 pipeline incorporated into QIIME2 (34). The DADA2 program filtered out phiX reads removed chimeric sequences and assigned reads into Amplicon Sequence Variants (ASVs) (35). Taxonomic annotation was obtained using the SILVA v138 database. Sequencing statistical analyses were done using QIIME2 v2023.2. Characterization, quantification and binning of ASVs was conducted with the analysis of the differential microbial distribution between the groups mentioned beforehand. Sequencing depth was studied with the rarefaction curve. Alpha (Chao1, Evenness, Observed features, Shannon, Simpson e, and Simpson) and beta (Principal Coordinate Analysis, Jaccard index, and Bray-Curtis) indexes were calculated to study diversity between groups. Venn diagrams were also generated.

### Statystical analyses

Descriptive statistics were calculated to describe the characteristics of the samples and groups. PERMANOVA test was used to compare beta diversity indexes, while ANOVA or Kruskal–Wallis (depending on the distribution of the variables) was used to compare other numerical variables between the three studied groups. Student's t-test or Mann–Whitney's *U*-test, with Bonferroni-Holm *p*-values adjustment, were applied to study differences between each pair of groups as *post-hoc* analysis. Kruskal–Wallis and chi-square tests were used to study differences between groups regarding abundance and presence of species/genus, respectively. Relevant results (*p* < 0.1) in these bivariate analyses were used to generate multivariate logistic regression models in order to study how the presence or abundance of different species/genius allowed differentiating samples from different groups.

## Results

### Participant characteristics and sequencing data summary

The clinical profile and clinicopathological information from each patient is shown in Table 1. The mean patient age was 66, 51 years (range 32−97), and 54.5% were males. 15 patients of the OL group and PVL group did not have dysplasia, 4 showed mild dysplasia, 11 moderate dysplasia, and 8 severe dysplasia. Most of the OSCC subjects were diagnosed at an early stage T1–T2 (64.3%) and presented with a lesion on the tongue (28.6%).

**Table 1:** Clinical and pathological information of the patients with OL, PVL and OSCC included in the study.

OL patients						
Case	Age	Gender	Location	Clinical type	Size (cm)	Histology
1	48	Male	Buccal mucosa	Homogeneous	3.0	Mild dysplasia
2	67	Male	Palate	Homogeneous	1.5	Epitelial hyperplasia
3	56	Female	Floor of the mouth	Homogeneous	2.4	Hyperkeratosis
4	88	Female	Tongue	Erythroleukoplakia	3.0	Severe dysplasia
5	60	Female	Floor of the mouth	Homogeneous	2.0	No dysplasia
6	44	Male	Floor of the mouth	Homogeneous	1.0	Mild dysplasia
7	50	Male	Tongue	Homogeneous	2.0	Hyperplasia
8	85	Male	Tongue	Erythroleukoplakia	2.5	Severe dysplasia
9	55	Female	Buccal mucosa	Homogeneous	4.1	Moderate dysplasia
10	76	Female	Tongue	Verrucous	2.1	Moderate dysplasia

PVL patients						
Case	Age	Gender	N. of locations	Locations	Clinical type	Histology
1	64	Male	3	Gingiva, buccal mucosa, tongue	Homogeneous, verrucous	Moderate dysplasia
2	47	Female	6	Gingiva, buccal mucosa, tongue, lips, floor of the mouth, palate	Homogeneous	Moderate dysplasia
3	63	Female	5	Gingiva, buccal mucosa, tongue, lips, palate	Homogeneous, verrucous	Verrucous hyperplasia
4	85	Male	4	Gingiva, buccal mucosa, tongue, palate	Homogeneous, verrucous	Severe dysplasia
5	45	Male	2	Gingiva, buccal mucosa	Homogeneous	Verrucous hyperplasia
6	71	Male	3	Gingiva, buccal mucosa, palate	Homogeneous, verrucous, ulcerative	Hyperkeratosis without dysplasia
7	77	Female	3	Gingiva, buccal mucosa, tongue	Homogeneous, verrucous	Verrucous hyperplasia
8	52	Female	4	Gingiva, buccal mucosa, tongue, palate	Homogeneous	Hyperplasia without dysplasia
9	66	Female	5	Gingiva, buccal mucosa, tongue, palate, floor of mouth	Homogeneous, verrucous, ulcerative	Moderate dysplasia
10	77	Female	3	Gingiva, buccal mucosa, palate	Homogeneous, ulcerative	Severe dysplasia
11	32	Female	3	Gingiva, buccal mucosa, palate	Homogeneous	Hyperplasia without dysplasia
12	69	Female	3	Gingiva, buccal mucosa, palate	Homogeneous	Severe dysplasia
13	69	Female	3	Gingiva, buccal mucosa, palate	Homogeneous	Hyperplasia without dysplasia
14	59	Male	3	Gingiva, buccal mucosa, palate	Homogeneous, verrucous	Moderate dysplasia
15	78	Male	3	Gingiva, tongue, floor of the mouth	Homogeneous, verrucous	Moderate dysplasia
16	70	Male	3	Gingiva, buccal mucosa, tongue	Homogeneous, verrucous	Severe dysplasia
17	54	Female	3	Gingiva, buccal mucosa, palate	Homogeneous, verrucous, ulcerative	Verrucous hyperplasia
18	62	Female	3	Gingiva, buccal mucosa, floor of the mouth	Homogeneous	Hyperplasia without dysplasia
19	70	Male	2	Gingiva, palate	Homogeneous, verrucous	Moderate dysplasia
20	67	Female	3	Gingiva, tongue and palate	Homogeneous, verrucous	Severe dysplasia
21	79	Female	5	Gingiva, buccal mucosa, tongue, floor of the mouth, palate	Homogeneous, verrucous	Moderate dysplasia
22	76	Female	4	Gingiva, buccal mucosa, lips, palate	Homogeneous, verrucous	Mild dysplasia
23	66	Female	4	Gingiva, buccal mucosa, tongue, palate	Homogeneous, verrucous, ulcerative	Moderate dysplasia
24	77	Female	3	Gingiva, buccal mucosa, palate	Homogeneous, verrucous	Moderate dysplasia
25	55	Male	4	Gingiva, buccal mucosa, tongue, floor of the mouth	Homogeneous, verrucous	Mild dysplasia
26	61	Female	5	Gingiva, buccal mucosa, tongue, floor of the mouth, lips	Homogeneous, verrucous	Severe dysplasia
27	76	Male	3	Gingiva, tongue, floor of the mouth	Homogeneous	Hyperkeratosis without dysplasia
28	66	Female	4	Gingiva, tongue, buccal mucosa, lips	Homogeneous, verrucous	Verrucous hyperplasia

OSCC patients					
Case	Age	Gender	Location	Clinical type	Staging
1	66	Male	Buccal mucosa	Ulcerative	Advanced (T3–T4)
2	70	Male	Tongue	Ulcerative	Advanced (T3–T4)
3	62	Male	Tongue	Ulcerative	Early (T1–T2)
4	58	Male	Palate	Ulcerative	Early (T1–T2)
5	82	Female	Tongue	Ulcerative	Advanced (T3–T4)
6	47	Male	Tongue	Ulcerative	Early (T1–T2)
7	54	Male	Buccal mucosa	Ulcerative	Early (T1–T2)
8	59	Male	Floor of the mouth	Ulcerative	Advanced (T3–T4)
9	61	Male	Gingiva	Ulcerative	Advanced (T3–T4)
10	53	Male	Tongue	Erythroleukoplakia	Early (T1–T2)
11	81	Male	Tongue	Ulcerative	Early (T1–T2)
12	66	Female	Tongue	Ulcerative	Advanced (T3–T4)
13	87	Male	Buccal mucosa	Ulcerative	Early (T1–T2)
14	78	Male	Floor of the mouth	Ulcerative	Advanced (T3–T4)
15	72	Male	Tongue	Exophytic	Early (T1–T2)
16	65	Female	Gingiva	Ulcerative	Early (T1–T2)
17	74	Male	Buccal mucosa	Ulcerative	Early (T1–T2)
18	64	Male	Floor of the mouth	Ulcerative	Early (T1–T2)
19	55	Male	Gingiva	Ulcerative	Early (T1–T2)
20	59	Male	Lips	Ulcerative	Advanced (T3–T4)
21	69	Male	Palate	Ulcerative	Early (T1–T2)
22	89	Male	Gingiva	Ulcerative	Early (T1–T2)
23	77	Male	Gingiva	Exophytic	Advanced (T3–T4)
24	97	Female	Lips	Ulcerative	Advanced (T3–T4)
25	74	Female	Floor of the mouth	Ulcerative	Early (T1–T2)
26	63	Female	Buccal mucosa	Ulcerative	Early (T1–T2)
27	87	Male	Palate	Ulcerative	Early (T1–T2)
28	59	Female	Gingiva	Ulcerative	Early (T1–T2)

Out of the 66 subjects, 2 samples were removed due to insufficient data quality. Sequencing of these samples yielded a total of 2,683,420 effective reads, with a median of 43,928.4 reads per sample (range 8,702–77,166). Of the 64 samples, 26 represent OSCC, 10 represent OL, and 28 represent PVL.

### Differences in microbiome diversity

**Figure 1:**
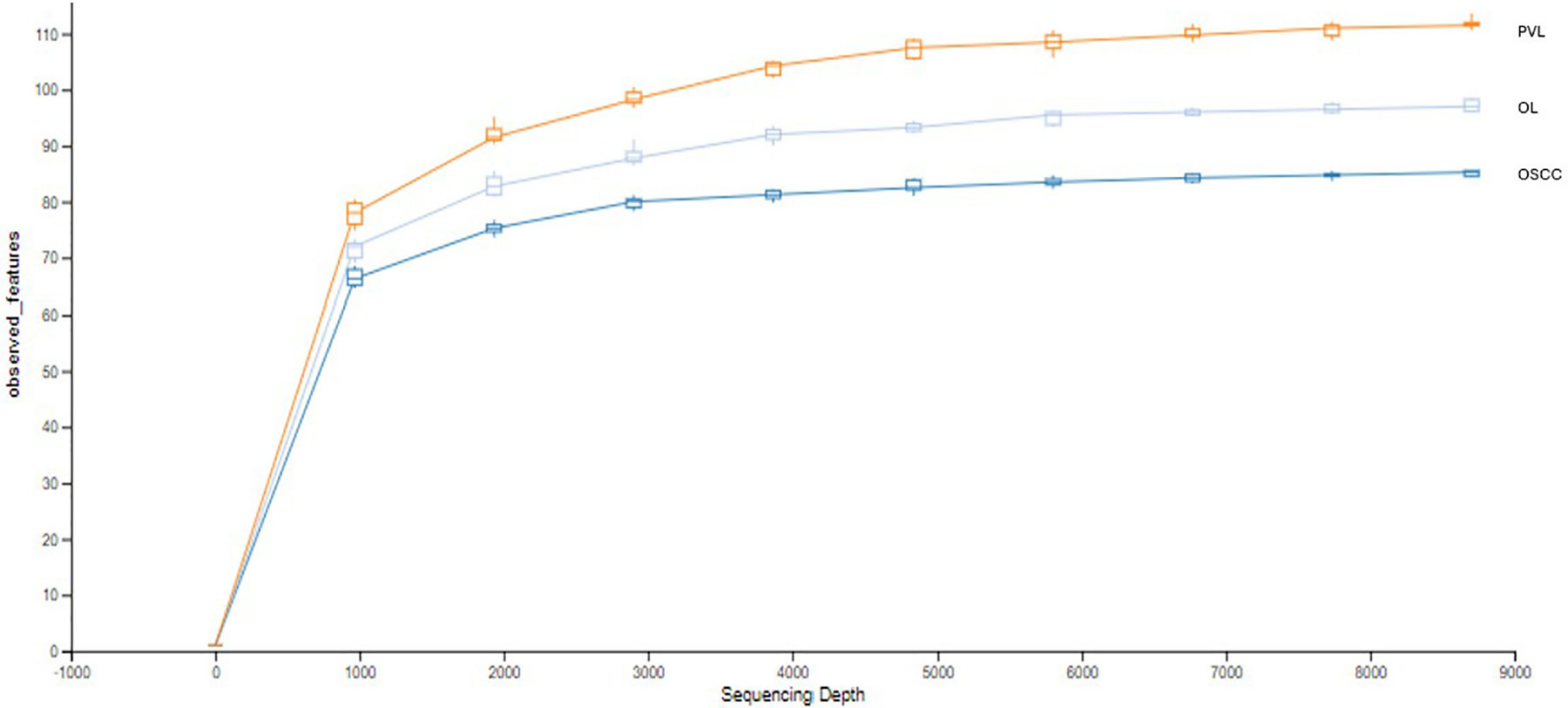
Rarefaction curve.

The rarefaction curve indicates the total richness within each group, with PVL being the richest, followed by OL, and then OSCC (Figure 1). The initial slope of each curve shows a rapid increase in the number of observed features with initial increases in sequencing depth suggesting high initial diversity, where many new features are discovered with each additional sequence. All the curves start to plateau as the sequencing depth increases, indicating that most of the microbial diversity has been captured.

**Figure 2:**
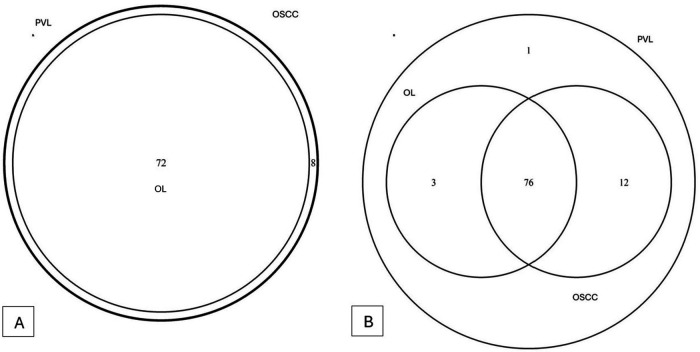
Venn diagram regarding genera differences **(A)** and species differences **(B)**.

**Figure 3:**
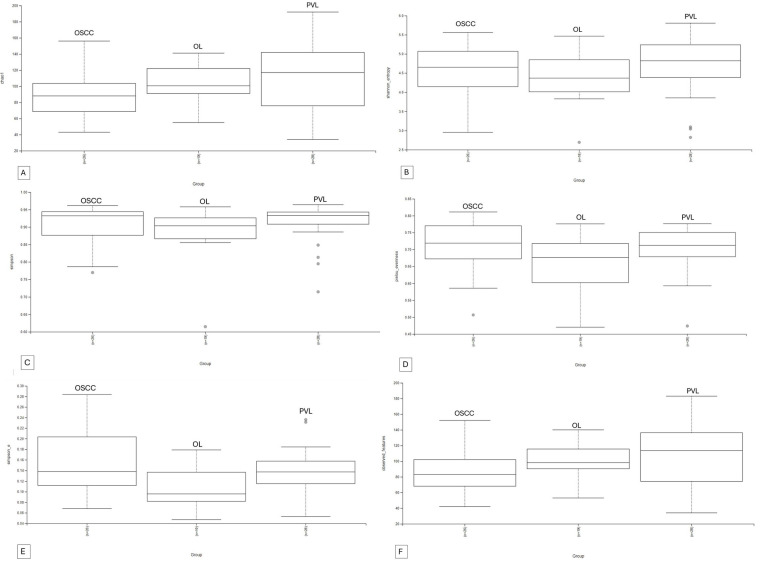
Alpha diversity indexes boxplots. **(A)**: Chao1, **(B)**: Shannon, **(C)**: Simpson, **(D)**: Evenness, **(E)**: Simpson E, **(F)** Observed Features.

A total of 11 phyla, 16 classes, 29 orders, 48 families, 80 genera and 98 species of bacteria could be detected. The overlapping regarding species and genera of the 3 groups can be appreciated in the Venn diagram (Figures 2A,B). Microbial alpha and beta diversity were evaluated. Several alpha diversity indices were assessed: Chao1, Shannon, Simpson, Evenness, Observed features, and Simpson e (Figures 3A–F). The analysis showed no statistically significant differences in alpha diversity between the groups (all *p*-values > 0.05) (Table 2).

**Table 2:** Kruskal–Wallis test results for alpha diversity indices across OSCC, OL, and PVL groups.

Alpha diversity metric	H Statistic	*p*-value
Chao1 index	2.650	0.266
Evenness index	3.524	0.172
Observed features	2.744	0.254
Shannon index	1.875	0.392
Simpson e index	4.469	0.107
Simpson index	2.131	0.345

**Figure 4:**
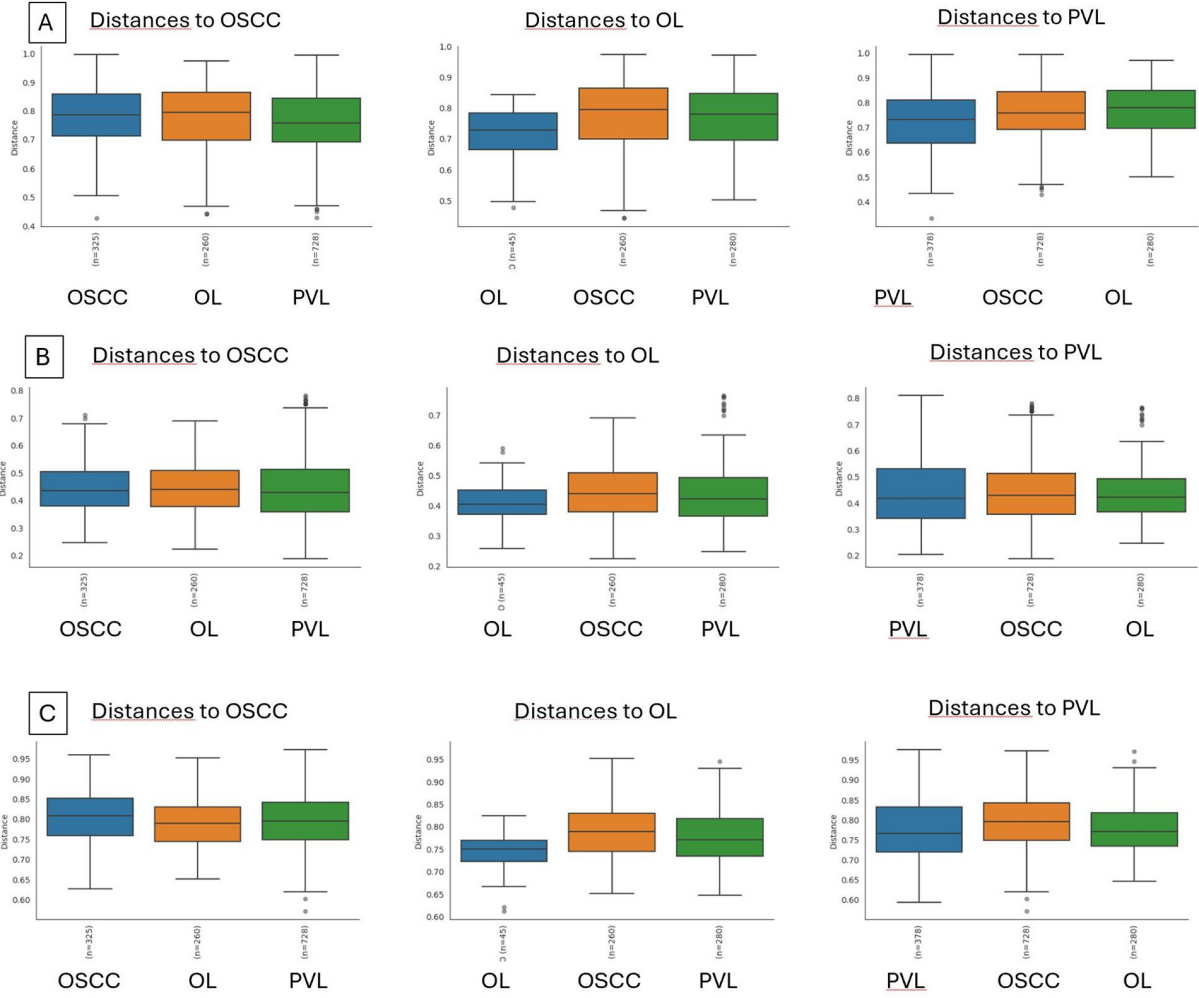
Beta diversity indexes boxplots. **(A)**: Bray-Curtis, **(B)**: Principal Coordinate Analysis (PCA), and **(C)**: Jaccard index.

Beta diversity was measured using different indexes: Bray-Curtis, Principal Coordinate Analysis (PCA), and Jaccard index (Figures 4A–C). All three indexes denote that there are significant differences in the microbial communities between OL and both PVL and OSCC, but there are no significant differences between PVL and OSCC (Tables 3).

**Table 3:** Combined pairwise PERMANOVA analysis (weighted PCA, jaccard, and bray-curtis indices).

Comparison	Sample Size	Weighted PCA pseudo-F (*p*-value, *q*-value)	Jaccard pseudo-F (*p*-value, *q*-value)	Bray-Curtis pseudo-F (*p*-value, *q*-value)
OSCC vs. OL	36	3.160932 (*p* = 0.021, *q* = 0.0315)	1.392923 (*p* = 0.021, *q* = 0.0315)	2.129558 (*p* = 0.003, *q* = 0.0075)
OSCC vs. PVL	54	2.099866 (*p* = 0.077, *q* = 0.0770)	1.177001 (*p* = 0.154, *q* = 0.1540)	1.270777 (*p* = 0.151, *q* = 0.1510)
OL vs. PVL	38	5.588071 (*p* = 0.005, *q* = 0.0150)	1.478415 (*p* = 0.020, *q* = 0.0315)	2.807113 (*p* = 0.005, *q* = 0.0075)

### Presence

According to *genus*, PVL presents all the 80 genera that has been detected. OSCC does not have Defluviitaleaceae_UCG-011; OL lacks Centipeda, [Eubacterium]_yurii_group, W5053, Ezakiella, Bacteroides, Comamonas, Anaerococcus, RF39 and Defluviitaleaceae_UCG-011.

According to *species*, PVL has the 92 detected species. Lachnospiraceae bacterium was detected only in PVL cases. OL does not have (but the other 2 groups do) Neissera oralis, Lactobacillus parabuchneri, Veillonella sp., Peptostreptococcaceae bacterium, Firmicutes oral, Prevotella nigrescens, Leptotrichia trevisanii, Prevotella heparinolytica, Ottowia sp., Leptotrichia buccalis, Leptotrichia goodfellowii. Regarding OSCC, besides not having Lachnospiraceae bacterium, the following were not detected: Porphyromonas endodontalis, Treponema denticola y Prevotella maculosa.

### Abundance

At the phylum level, the most abundant bacterial groups detected with values above 5% were as follows: in OSCC, Firmicutes (67%), Actinobacteriota (8.5%), and Fusobacteriota (6.4%); in OL, Firmicutes (64.1%), Actinobacteriota (16.5%), and Patescibacteria (5.5%); and in PVL, Firmicutes (49.3%), Proteobacteriota (13.6%), Bacteroidota (12.5%), and Actinobacteriota (10.4%). A significant difference was found in Actinobacteriota (*p* = 0.002), with OL exhibiting significantly higher levels compared to OSCC and PVL (Table 4).

**Table 4:** Phylogenetic composition of the most common taxa by group.

Phylum	Genus	OSCC (%)	HL (%)	PVL (%)	*χ*^2^ Test	*p*-value
Firmicutes		67 (40.2–88.4)	64.1 (47.4–83.8)	49.3 (33.5–94.6)	8.826	0.012
	*Streptococcus*	36 (12.2–68.2)	48.6 (21.7–75.2)	27.2 (12.1–81.2)	9.261	0.01
	*Veillonella*	9.2 (0.8–32.0)	1.9 (0.1–9.6)	8.4 (0.3–29.7)	9.547	0.008
	*Granulicatella*	1.5 (0.0–9.9)	2.1 (0.3–5.8)	1.6 (0.0–6.7)	0.091	0.956
	*Gemella*	3.5 (0.0–11.7)	2.1 (0.3–4.7)	3.3 (0.0–6.3)	1.577	0.455
	*Parvimonas*	0.7 (0.0–23.7)	1.2 (0.1–6.4)	0.6 (0.0–4.5)	0.976	0.614
	*Lactobacillus*	0.1 (0.0–30.2)	0.0 (0.0–0.9)	0.0 (0.0–24.5)	2.831	0.243
	*Oribacterium*	0.7 (0.0–5.8)	0.2 (0.1–2.1)	0.4 (0.0–2.5)	3.163	0.206
	*Solobacterium*	0.7 (0.0–6.5)	1.1 (0.1–5.1)	0.4 (0.0–3.0)	3.101	0.212
	*[Eubacterium] brachy group*	0.1 (0.0–1.4)	0.1 (0.0–4.4)	0.1 (0.0–1.8)	1.358	0.507
Actinobacteriota		8.5 (0.0–24.8)	16.5 (10.8–27.5)	10.4 (1.0–38.8)	12.171	0.002*
	*Rothia*	4.3 (0.0–19.6)	10.0 (1.6–24.1)	7.7 (0.1–34.3)	7.954	0.019
	*Atopobium*	0.8 (0.0–3.2)	1.3 (0.1–11.3)	0.8 (0.0–3.7)	1.621	0.445
	*Actinomyces*	0.7 (0.0–8.9)	2.5 (0.5–7.9)	0.4 (0.0–4.6)	13.376	0.001*
Protebacteria		4.2 (0.0–54.8)	0.9 (0.0–2.2)	13.6 (0.0–36.7)	8.595	0.014
	*Haemophilus*	0.9 (0.0–37.4)	0.0 (0.0–6.4)	4.5 (0.0–16.0)	10.911	0.004
	*Neisseria*	1.7 (0.0–43.1)	0.2 (0.0–12.3)	4.9 (0.0–22.4)	5.269	0.072
Patescibacteria		2.4 (0.0–15.5)	5.5 (1.2–14.7)	2.3 (0.0–12.4)	5.087	0.079
	*TM7x*	1.0 (0.0–13.2)	2.0 (0.1–13.9)	0.5 (0.0–11.9)	2.309	0.315
	*Saccharimo-nadaceae*	0.1 (0.0–11.3)	1.9 (0.0–10.5)	0.4 (0.0–3.9)	6.686	0.035
Bacteroidota		4.6 (0.2–23.5)	2.0 (0.2–10.2)	12.5 (0.0–25.7)	4.476	0.107
	*Porphyromonas*	1.0 (0.0–17.1)	0.2 (0.0–5.0)	3.6 (0.0–24.2)	9.356	0.009
	*Prevotella*	0.5 (0.0–7.0)	0.4 (0.0–8.7)	0.8 (0.0–11.5)	0.303	0.86
	*Capnocytophaga*	0.5 (0.0–6.5)	0.3 (0.0–1.9)	1.2 (0.0–9.0)	1.618	0.445
Fusobacteriota		6.4 (0.1–30.6)	0.8 (0–5.4)	3.8 (0–14.3)	3.371	0.185
	*Leptotrichia*	2.8 (0–30.6)	0.4 (0–10.9)	3 (0–13.8)	4.513	0.105
	*Fusobacterium*	0.6 (0–9)	0.2 (0–10.7)	0.8 (0–3.2)	0.94	0.625

Abundance at the phylum and genus levels are presented as the median percentage (with lowest and highest value) of the total microbiome.

* *p* < 0.05.

At the genus level, considering taxa with median abundances above 1%, OSCC presented Streptococcus (36.0%), Veillonella (9.2%), Rothia (4.3%), Gemella (3.5%), Leptotrichia (2.8%), Neisseria (1.7%), Granulicatella (1.5%), TM7× (1.0%), and Porphyromonas (1.0%). In OL, the dominant genera included Streptococcus (48.6%), Rothia (10.0%), Actinomyces (2.5%), Granulicatella (2.1%), Gemella (2.1%), TM7× (2.0%), Veillonella (1.9%), Saccharimonadaceae (1.9%), Atopobium (1.3%), Parvimonas (1.2%), and Solobacterium (1.1%). PVL showed a composition of Streptococcus (27.2%), Veillonella (8.4%), Rothia (7.7%), Neisseria (4.9%), Haemophilus (4.5%), Porphyromonas (3.6%), Gemella (3.3%), Leptotrichia (3.0%), Granulicatella (1.6%), and Capnocytophaga (1.2%).

A statistically significant difference was found in Actinomyces, with OL showing higher levels than OSCC and PVL (*p* = 0.001). Further pairwise comparisons and multivariate analyses provided additional insights regarding presence and abundance separately (Table 5). Comparing the presence of OSCC with OL, the genera Saccharimonadales and F0332 were significantly associated with OL cases (*p* = 0.024 and *p* = 0.01), while Rothia abundance showed an almost significant tendency, suggesting that higher levels correlate with a greater probability of OL (estimate = 0.205; *p* = 0.052). When comparing the abundance between OSCC with PVL, a statistically significant association was found for Rothia, indicating that increased abundance was linked to PVL cases (*p* = 0.01). In the comparison between OL and PVL abundance, Veillonella and Porphyromonas exhibited an almost significant trend, suggesting that their higher abundance may be associated with PVL (estimate for Veilonella = 0.27 and *p* = 0.09; estimate for Porphyromonas = 0.76 and *p* = 0.09).

**Table 5:** Presence and abundance coefficients per genus and species.

Presence coefficients	Genus	Comparison	Estimate Std.	Error	*Z* value	Pr (>|z|)
	*Saccharimonadales*	OSCC vs. OL	2.897	1.290	2.245	0.0248*
	*F0332*	OSCC vs. OL	3.373	1.440	2.343	0.0191*
Presence coefficients	Species	Comparison	Estimate Std.	Error	*Z* value	Pr (>|*z*|)
	*Leptotrichia trevisanii*	OSCC vs. PVL	−3.591	1.671	−2.149	0.0316*
	*Bifidobacterium longum*	OSCC vs. PVL	−2.520	1.525	−1.653	0.0984
	*Bifidobacterium longum*	OL vs. PVL	−2.59	1.475	−1.762	0.07808
Abundance coefficients	Genus	Comparison	Estimate Std.	Error	*Z* value	Pr (>|*z*|)
	*Rothia*	OSCC vs. OL	0.205	0.105	1.947	0.0515
	*Rothia*	OSCC vs. LVP	0.139	0.058	2.371	0.0177*
	*Veillonella*	OL vs. LVP	0.273	0.163	1.676	0.0938
	*Porphyromonas*	OL vs. LVP	0.767	0.456	1.679	0.0931
Abundance coefficients	Species	Comparison	Estimate Std.	Error	*Z* value	Pr (>|*z*|)
	*Candidatus Saccharibacteria*	OSCC vs. OL	4.065	2.277	1.785	0.07426

*p < 0.05.

At the species level, considering taxa with median abundances above 1%, OSCC was dominated by Streptococcus salivarius (12.6%), Streptococcus parasanguinis (7.8%), and Schaalia odontolytica (1.9%). In OL, Streptococcus salivarius (25.6%), Streptococcus parasanguinis (10.9%), and Schaalia odontolytica (5.7%) were the most abundant species. PVL was characterized by Streptococcus salivarius (12.4%), Streptococcus parasanguinis (5.1%), Neisseria perflava (4.6%), Prevotella melaninogenica (2.2%), Capnocytophaga gingivalis (1.1%), and Schaalia odontolytica (1.0%).

There were not statistically significant results with the Kruskal–Wallis test between groups regarding species. We generated multivariate logistic regression comparing pairs of groups with all the variables that generated statistically significant results when comparing pairs of groups. The analysis involved both the presence and abundance separately (Table 5). When comparing the abundance between OSCC with OL, Candidatus Saccharibacteria showed a low *p*-value (not statistically significant), but its higher abundance tended to be associated with OL cases (estimate = 4.06 and *p* = 0.07). In the OSCC vs. PVL comparison regarding presence, Leptotrichia trevisanii was significantly associated with OSCC (*p* = 0.03), while Bifidobacterium longum showed an almost significant trend in the same direction (estimate = −2.5 and *p* = 0.09). In the OL vs. PVL comparison regarding presence, Bifidobacterium longum displayed an almost significant result, suggesting that its presence is associated with OL cases (estimate = −2.59 and *p* = 0.07).

## Discussion

In this study, we characterized the oral microbial communities of patients with OL, PVL and OSCC. In terms of overall diversity, as indicated by the rarefaction curve, PVL demonstrated to have the highest richness, while OSCC showed the lowest values. A richer, more diverse microbiome, characterized by a higher number of reads and OTUs, is commonly observed in healthy individuals. In contrast, disease conditions are often linked to decreased richness and diversity within the microbiome. Guerrero-Preston et al. underscored this pattern specifically in OSCC cases, where the total bacterial OTUs in OSCC samples numbered 3,659, compared to 13,849 in healthy individuals (36). The decrease in oral microbial diversity has been described previously in different cancers, including oral, esophageal and nasopharyngeal (37–39). This decrease in diversity in OSCC could be interpreted as part of the disease progression, where cancer-induced changes in the oral environment might lead to a selective reduction in microbial richness. In line with Herreros-Pomares' findings, OL and PVL in our study did not show a significant decrease in microbial diversity when compared to OSCC (40). Both premalignant lesions maintained a relatively higher number of observed features, which may suggest that microbial dysbiosis is more pronounced in fully malignant lesions like OSCC. However, reduction in oral microbial diversity has been described in other oral diseases such as recurrent aphthous stomatitis and oral lichen planus and caution must be taken when interpreting these data (37, 41, 42). In fact, it is still unclear whether the microbial alterations detected contribute to the onset of the disease or are a consequence of the disease process. Without longitudinal data, it's ardous to determine whether microbial changes occurred before the disease or after it. Two main theories have been suggested. The first, known as the “bacteria before tumor” hypothesis, proposes that bacterial-induced damage to epithelial cells triggers an inflammatory cascade, leading to increased cell replication, production of reactive oxygen species (ROS), and ultimately DNA damage that contributes to carcinogenesis. The second theory, termed “bacteria after tumor,” suggests that opportunistic bacteria are drawn to the hypoxic and highly vascularized tumor environment, where they support the progression of the diseased ecosystem (43).

A few studies have investigated alpha diversity between leukoplakia patients and healthy subjects, reporting either richness, depletion or equivalence between the 2 groups; beta diversity has also been reported with difficulty in interpreting the results given the different subsite or subgroup analyses (44). In our study, alpha diversity did not show statistically significant results for the 3 groups, but beta diversity showed that the microbial communities in OL are different from those in PVL and OSCC. This concept ratifies that oral leukoplakia is indeed a distinct entity from PVL and not only due to its clinical presentation and evolution/prognosis, but as suggested by our study, due to the microbiome as well. In a recent investigation by Herreros-Pomares et al., a methylation analysis was performed between patients with OL and patients with PVL and showed differential methylation patterns existing between the 2 entities, further confirming their diverse nature (45).

The literature provides valuable insights into the core oral microbiome composition across individuals. Verma et al. highlight that in healthy conditions, six main phyla dominate the oral cavity: Firmicutes, Actinobacteria, Proteobacteria, Fusobacteria, Bacteroidetes, and Spirochaetes (46). In our study, we also found the presence of Patescibacteria in all 3 groups, with predominance in OL. The role of Patescibacteria remains largely speculative but is of growing interest. Their abundance and potential interactions in dysbiotic states or diseases have not been fully delineated, and their precise functions within microbial communities are still under investigation. A study by Hu et al., analyzed the relationship between various microbiome taxa and esophageal cancer. Their findings suggested that certain phyla, including *Patescibacteria*, may have a protective effect against this type of cancer. The study highlighted the potential indirect roles that microbial populations might play in cancer progression, though it emphasized that further research is needed to understand these associations (47). Herreros-Pomares detected a an abundance of Campylobacterota of >10% in PVL and PVL-OSCC patients, which was found to be associated to and increased risk of esophageal and oral cancer in other studies (40). However, in our study it was not remarkable for either groups.

In our investigation, OL had significantly higher levels of Actinobacteriota compared to the other 2 groups. This could indicate that these bacteria could have a role in early-stage lesions or in maintaining a premalignant status. Amer et al., observed a decrease in the abundance of Firmicutes and an increase in the abundance of Fusobacteria and Actinobacteria in swabs from leukoplakia samples compared to healthy mucosa from the same patients (6). Gopinath showed increased Bacteroidetes and reduced Firmicutes in OL samples; while in the study by Hu et al., OL were associated with reduced Firmicutes and increased Bacteroidetes (30, 48). At the genus level, Bik et al. observed Actinomyces, Atopobium, Corynebacterium, Rothia of Actinobacteria; Bergeyella, Capnocytophaga, Prevotella of Bacteroidetes, Granulicatella, Streptococcus, Veillonella of Firmicutes, Fusobacterium, Campylobacter, Cardiobacterium, Haemophilus, Neisseria of Proteobacteria, and TM7 as main components of the oral microbiome (49). Specifically, OL was enriched with Streptococcus, Rothia, Actinomyces, Granulicatella, Gemella, TM7x, Veillonella, Saccharimonadaceae, Atopobium, Parvimonas, Solobacterium; PVL had a higher abundance of Streptococcus, Veillonella, Neisseria, Haemophilus, Porphyromonas, Gemella, Leptotrichia, Granulicatella and Capnocytophaga and OSCC was enriched with Streptococcus, Veillonella, Rothia, Gemella, Leptotrichia, Neisseria, Granulicatella, Porphyromonas.

Our study showed that OL cases presented statistically significant results for Actinomyces, suggesting that Actinomyces may be a distinguishing factor in the microbial landscape of OL, potentially contributing to its unique pathophysiology.

Amer et al. detected that the microbiome of oral leukoplakia exhibits enrichment for Fusobacterium, Leptotrichia, Campylobacter, and Rothia which bears similarity to recently identified enrichments on colorectal carcinoma (6).

Eubacterium, Porphyromonas and Tannarella were more abundant in PVL cases compared to controls in Herreros-Pomares study; specifically, Eubacterium has been considered a presumed periodontitis pathogen while Tannarella and Porphyromonas have been related to periodontitis and Head and neck cancer (21). Herreros-Pomares also found that PVL cases were enriched in Aggregatibacter, which has been associated with aggressive periodontitis and has been suggested to modulate the immune response through leukotoxin and LPS (21).

A study by Gopinath et al., which compared salivary samples in patients with OL, OSCC and healthy subjects showed that the controls had abundance in Megaspheara, unclassified enterobacteria, Prevotella, Porphyromonas, Rothia, Salmonella, Streptococcus, and Fusobacterium (48). In another study, OSCC were enriched in Capnocytophaga, Fusobacterium, Leptotrichia, Neisseria, Bergeyella, Mycoplasma, Johnsonella, and Staphylococcus; and PVL-OSCC in Selenomonas, Catonella, and Defluviitaleaceae UCG−011 (40). In particular, Capnocytophaga has been described to be a potential tumor promotors and potential microbiome marker in oral cancer (40).

Ganly et al. found an enrichment of Fusobacterium, Prevotella and Alloprevotella but depletion of Streptococcus along the sequence “controls → OL → OSCC” and reported Fusobacterium and Veillonella to be more abundant in OL compared to healthy controls (50). We also describe an increase of Fusobacterium and a decrease of Streptococcus in OSCC samples compared to OL. Gopinath found reduced levels of Granulicatella and Porphyromonas gingivalis in OL compared to OSCC (48). Hashimoto et al. observed an increased abundance of Porphyromonas gingivalis and Streptococcus anginosus in OL and OSCC relative to the controls (51). The genus Streptococcus, which represents the main oral commensal, when depleted is often reported in regard to premalignant and malignant lesions (44). We also observed a diminished trend of Rothia when comparing OL to OSCC.

In terms of species, Streptococcus Salivarius and Streptococcus Parasanguinis were the most common through the three groups. Streptococcus Parasanguinis was highly associated with tumor site compared to non-tumor site in one study (52). In another study, Streptococcus Parasanguinis was higher in tongue and pharyngeal subjects (53). Herreros-Pomares found a higher abundance for the OL group regarding Streptococcus Parasanguinis and Prevotella Histicola (40).

In our study, there was an increased abundance for OL compared to PVL regarding Schaalia Odontolytica. Schaalia Odontolytica, which is part of Actinomyces, has been described in bloodstream infections and actinomycotic lesions (54). Bifidobacterium Longum, has shown efficacy in improving irritable bowel syndrome, and in suppressing colorectal cancer through the modulation of intestinal microbes and immune function (55, 56). In the study conducted by Amer et al., Rothia mucilaginosa, Alloprevotella sp., Neisseria meningitides, and Leptotrichia sp. significantly elevated and Neisseria oralis, Streptococcus infantis, and Lautropia mirabilis significantly diminished in oral leukoplakia compared to control (6). Another study, showed that Haemophilus was significantly elevated and Bacillus and Abitrophia significantly decreased in leukoplakia patients compared to healthy subjects (30). Neisseria oralis was not present in our OL group. Amer et al. noticed that Neisseria oralis, Streptococcus infantis, and Lautropia mirabilis significantly diminished in oral leukoplakia compared to control (6).

Our PVL group, besides Streptococcus Salivarius and Streptococcus Parasanguinis, was also enriched in Neisseria perflava, Prevotella melaninogenica, Capnocytophaga gingivalis, Schaalia odontolytica. Capnocytophaga gingivalis has been described to be a potential tumor promoter in oral cancer, and it has found that its supernatant induced OSCC cells to undergo EMT, causing the cells to acquire a mesenchymal phenotype associated with highly invasive and metastatic properties (57). Herreros-Pomares reported that compared to OL, PVL patients were enriched in Campylobacter concisus, Prevotella salivae, and Dialister pneumosintes (40). In another study, Herreros-Pomares also found abundance of Oribacterium sp. Oral taxon 108, which has been described to be more abundant in saliva samples of Acute Leukemia patients compared to controls, and Campylobacter jejuni, which has been described to promote colorectal tumorigenesis through DNA damage (21).

In our study, PVL was the only group presenting with Lachnospiraceae bacterium. This species was also present in abundance in PVL-OSCC cases in Herreros-Pomares study (40).

Comparing OSCC with PVL in our study, the presence of Leptotrichia trevisanii is associated with a higher probability of being a case of OSCC. In the study conducted by Lyu et al., Leptotrichia trevisanii, Capnocytophaga gingivalis, Leptotrichia buccalis, Peptostreptococcus anaerobius, Streptococcus pneumoniae, and Campylobacterureolyticus were significantly increased in tumor non-recurrent compared to tumor recurrent samples (58).

Herreros-Pomares, found that in OSCC patients, Capnocytophaga leadbetteri, Capnocytophaga sputigena, and Capnocytophaga gingivalis had a higher abundance and Metamycoplasma salivarium and Prevotella nanceiensis were also elevated (40).

In the literature, two bacteria, Fusobacterium nucleatum and Porphyromonas gingivalis, have been shown to support carcinogenesis in a murine model (44). Metatranscriptomic analysis of the microbiome associated with OSCC, or “oncobiome,” has revealed specific bacterial activities, including elevated metal ion transport, nitrous oxide reductase, and heightened tryptophanase and protease activity. Increased ion transport around cancer sites is linked to the catalysis of radical ions and the stimulation of cancer cell growth. Additional functions of the OSCC-associated microbiome include anaerobic respiration, proteolysis, and defense mechanisms against oxidative stress and radical species damage (43).

One limitation in our study, is the absence of a healthy control group which could help further clarify whether the reduced diversity seen in OSCC is indeed a consequence of the cancer process or reflects a broader trend in oral dysbiosis. Despite the lack of a healthy subjects, the diversity differences observed between OL, PVL, and OSCC still provide valuable insights into how microbial richness changes as oral lesions progress toward malignancy. Future directions may involve designing a longitudinal study that tracks microbial changes over time in order to monitor microbial shifts as lesions progress or regress, shedding light on whether specific taxa or microbial patterns precede disease development or are a consequence of it. Additionally, future studies might stratify data by factors like clinical and histopathological differences like the grading of dysplasia or differences between homogenous and non-homogeneous leukoplakia, as non-homogenous leukoplakia has been described to harbor more probability to progress into malignancy and lifestyle covariates like smoking, alcohol use, diet, oral hygiene practice, and geographical environment. Finally, collecting saliva might represent a broad microbial composition, given the fact that the oral cavity is a complex ecosystem with different niches. It is unlikely that the disease is caused by a single bacterium; rather, it likely involves clusters of bacteria with complex interactions that can be either beneficial or harmful. Future studies might involve collecting samples from multiple sites rather than relying on a single sample. While 16S rRNA sequencing provides taxonomic insights, it does not reveal the functional potential of the microbiome. In cancer and OPMDs, certain bacterial functions (e.g., inflammation induction, carcinogen metabolism) could play a crucial role. Future directions might integrate metatranscriptomics to investigate microbial genes and pathways involved in carcinogenesis, helping to understand not only “who is there” but also “what they are doing.”

## Conclusions

Using a 16S rRNA gene-sequencing based approach, we laid the foundation for defining the microbial signature of patients with OL, PVL, and OSCC. PVL exhibited the highest species richness, suggesting a more diverse microbiome composition, while OSCC showed the lowest richness. These findings align with the understanding that reduced microbial diversity often accompanies malignancy, where a more selective environment fosters specific microbial growth, potentially aiding carcinogenesis. Significant differences were observed between OL and both PVL and OSCC, highlighting a distinct microbial community in OL compared to more malignant conditions. Notably, the lack of significant difference between PVL and OSCC suggests that these conditions may share microbial community traits as part of a progressive shift towards malignancy. Specifically, OL showed to have different microbiota compared to PVL and OSCC as demonstrated by the statistically significant result for Actinomyces. This finding supports the previous investigations showing that OL and PVL are actually different entities due to their different clinical aspects, evolution, and methylation patterns. PVL represents indeed an OPMD one step closer to OSCC and deserves strict monitoring as already suggested by previous studies. A better understanding of the role of the microbiome in future studies could provide precious information regarding non-invasive diagnostic and prognostic alternatives, as well as targeted therapeutic interventions.

## Data Availability

The datasets presented in this study can be found in online repositories. The names of the repository/repositories and accession number(s) can be found in the article/Supplementary Material.
